# Perceived Stress Scale (PSS-10) psychometric properties in migrants and native Germans

**DOI:** 10.1186/s12888-020-02851-2

**Published:** 2020-09-11

**Authors:** Christina Diane Bastianon, Eva M. Klein, Ana Nanette Tibubos, Elmar Brähler, Manfred E. Beutel, Katja Petrowski

**Affiliations:** 1 Medical Psychology and Medical Sociology, University Medical Center of the Johannes Gutenberg, University Mainz, Mainz, Germany; 2 Department of Psychosomatic Medicine and Psychotherapy, University Medical Center of the Johannes Gutenberg University Mainz, Mainz, Germany

**Keywords:** PSS, Psychometric properties, Stress, Migration, Measurement invariance

## Abstract

**Background:**

With the increasing diversity of the German population, it is important to test the psychometric validity and reliability of the German version Perceived Stress Scale (PSS-10) specifically between German natives and residents with a migration background.

**Methods:**

Using nationally representative data (*N* = 2527), this study conducted an Exploratory Factor Analysis (EFA) to determine the most appropriate factor structure, a Multi-Group Confirmatory Factor Analysis (MGCFA) to compare the validity of the two-factor structure and tested the PSS-10 measurement invariance between the German native and migrant sub-samples. Lastly, reliability of the PSS-10 was examined via Cronbach’s alpha, omega and individual item analyses across the two sub-samples.

**Results:**

The EFA results support a two-factor structure in the migrant sample. The MGCFA showed adequate model fit for both sub-samples and the PSS-10 is strict invariant between German natives and migrants. Cronbach’s alpha and omega for *Perceived Helplessness* (PHS: factor 1) and *Perceived Self-Efficacy* (PSES: factor 2) demonstrate good internal consistency in both German and migrant sub-samples.

**Conclusions:**

The key conclusions are: (1) the German version PSS-10 is suitable for German residents with a migration background. (2) Despite good internal consistency for the total scale, the PSS-10 measures two aspects: (a) perceived helplessness and (b) perceived self-efficacy. Future research would profit from analyzing the two subscales separately, not only using the total score.

## Background

Mental health and specifically stress have gained attention as public health concerns by the European Regional Office of the World Health Organization (WHO) and by the German national government. Objective 1 of *The European Mental Health Action Plan 2013–2020* [1] focuses on equal opportunities for mental health well-being, specifically recognizing vulnerable or at risk groups. The *Stress Report Germany 2012* [2] echoes the importance of bringing attention to and researching the impacts of stress and mental health, especially in the ever-changing work environment. The S*tress Report Germany 2012* [2] found that perceived stress and the number of health complaints increased from the 2005/2006 report. Most importantly, both the WHO European Regional Office and *Stress Report 2012* emphasize the inequality of how stress and mental health impact various groups, particularly vulnerable and disadvantaged groups which may be characterized by ethnicity, sex, age, religion, sexuality, refugee or immigrant status, socioeconomic status and physical and/or mental disability [1, 2].

Germany has a rich immigration history that has led to the diversity of its population. Work-visa programs from the mid-1950s until 1973 explain, in large part, the earlier settlement of Italians and Spaniards, but also the Turkish, Polish and Romanian populations within Germany [3, 4]. In more recent years, immigration flows consisted not only of economic migrants, but increasingly people fleeing persecution and war-torn countries. The total foreign population in Germany in 2014, including EU28, is reported at just over 8 million [5]. Citizens of Turkey (11.2%), Poland (8.6%), Syria (7.1%), Romania (6.9), and Italy (5.1%) represent the highest percentages of the foreign-born population of Germany in 2018 [6].

Migrants (first- and second-generation) have long been a focus of stress research. Regardless of whether migration occurred voluntarily or as a result of natural disasters or displaced by conflict, many aspects of the migration process are seen as stressors [7]. Even after settling in a new country, those who have migrated may still experience stress as they adapt to their new home through acculturation and integration stressors [8]. In Russian and Iranian migrants in Germany, Haasen, Demiralay, and Reimer [9] found a significant correlation between acculturative stress and mental distress; the length of residency in Germany did not have a significant effect. Therefore, it is important to take a closer look at stress within the population of residents with a migration background [10]. However, it is not known, if generalized mental health or stress are also responsive to migrant specific stressors such as integration, discrimination, acculturation or PTSD. Therefore, it is important in advancing stress research to test whether widely used scales maintain psychometric strength and measurement equivalence in migrant specific populations.

Within the German population, only a few studies exist testing the measurement equivalence of mental stress measures between populations with and without a migration background. Tibubos et al. [11] demonstrated strict measurement invariance of the Patient Health Questionnaire-9 between migrant status (nonimmigrants, 1st generation migrants and 2nd generation migrants) and country of origin in a German cohort study, the Gutenberg Health Study (GHS), with 13,973 participants. In another study with *N* > 26,000 participants from the German Socio-economic Panel (SOEP) including nonimmigrant, migrant and refugee populations in Germany from 16 different countries, the Patient Health Questionnaire-4 and the Short Form Health Survey (SF-12) showed scalar invariance (1) between men and women, (2) between groups stratified by migration status, (3) between survey languages, (4) between country of origin, (5) between sex and country of origin, and (6) between age groups [12].

The Perceived Stress Scale developed by Cohen, Kamarck and Mermelstein [13] is a widely used self-report measure assessing “the degree to which situations in one’s life are appraised as stressful”(p.387). The scale measures, over the past month, the degree to which life has been experienced as unpredictable, uncontrollable and overloaded. The original scale included 14 items but was later reduced to 10 items due to low factor loadings on 4 items; this change marginally improved the scale reliability shown via Cronbach’s alpha [14]. An additional 4 item version was developed for telephone interviews or situations with time restrictions [14], however, this short form has not fared as well as the full 14- and 10-item versions [15]. In a review of the PSS psychometric properties, Lee [15] showed across the 19 studies included, the PSS-10 was found to be superior to the 14-item version. Cronbach’s alpha constantly surpassed the standard .70 threshold ranging between .74–.91 [15]. The review also found consistent results supporting a two factor structure, which is in contrast to the original one-factor structure presented by Cohen et al. [13]. This debate was sparked after Hewitt, Flett and Mosher [16] challenged the one-factor structure, recognizing that both factors explained unique variances of depression and that factor 1 comprised of ‘adaptational symptoms’ while factor 2 reflected ‘coping ability’. Roberti, Harrington and Storch [17] further supported the two-factor structure naming factor 1 *perceived helplessness* (PHS) and factor 2 *perceived self-efficacy* (PSES). The 10-item version has proven to be a valuable tool for stress research as it maintains consistent test-retest reliability across various timespans, acceptable Cronbach’s alpha, and strong factorial validity in measuring perceived stress across various populations and languages [18–22]; see review: [15]. Although the PSS-10 has been used in specialized populations, in particular minority groups, many psychometric studies on the PSS-10 call for continual testing in more diverse and representative populations [15, 18].

In a representative German population, Klein et al. [23] tested the translated German version PSS-10 showing a strong Cronbach’s alpha = .84, further emphasizing the strength of the scale’s internal consistency. Reis and colleagues [24] tested the factor structure of the German version PSS-10 using bifactor modeling bringing a deeper understanding to the multidimensionality of the scale. Most recently, Schneider et al. [25] tested the PSS-10 two-factor structure between clinical and nonclinical samples, showing strict measurement invariance. Although the PSS-10 has been translated and tested in numerous languages, populations and contexts, the psychometrics of the German version PSS-10 have not been tested for migrant populations within Germany. Considering the popularity of the PSS-10 as a generalized measure of stress and its various applications, this study aims to fill this gap by testing the validity, reliability, and measurement equivalence of the German version PSS-10 between native Germans and migrants.

## Methods

### Data collection

This study, including the consent procedure, was approved by the institutional ethics review board of the University of Leipzig (Az 063–14-10,032,014). Furthermore, the study adhered to ICH-GCP-guidelines along with the ICC/ESOMAR International Code of Marketing and Social Research Practice. All participants were informed of the study procedures, data collection and anonymization of all personal data. According to German law, all participants provided verbal informed consent, which was noted by the interviewer before starting with the survey.

This study uses data from a representative survey of the German population and the same data as in Klein et al. [23]. Data were collected by USUMA (Unabhängiger Service für Umfragen, Methoden und Analysen; Berlin) between February and April 2014. The sample consisted of a total of *N* = 2527 participants between the ages of 14 and 95 years old (detailed sample description is found below in Table 1). Face-to-face interviews were conducted by trained interviewers via a stratified, random-route procedure in line with the ADM (Arbeitskreis Deutscher Markt- und Soziolforschungsinstitute e.V.) sampling guidelines. Questionnaires were independently completed by the participant in the presence of the interviewer.

**Table 1:** Descriptive Characteristics

		Natives	Migrant	t-test	*p*
		*N* = 2195	*N* = 328		
		87%	13%		
Gender	Male	46.33	48.48	0.72	0.46
	Female	53.67	51.52		
Age	Mean	49.86	46.46	3.23	0.001***
	SD	17.74	18.19		
	Range	14–95	14–86		
Marital Status	Single	27.47	25.91	2.48	0.01**
	Married	45.15	52.44		
	Separated^a^	2.28	0.91		
	Divorced	13.90	13.72		
	Widowed	11.12	7.01		
Education	Less than 10	36.99	47.56	1.30	0.19
In years	10–13	50.48	37.50		
	Greater than 13	10.11	10.6		
	Other	2.41	4.88		
Employment	Full-time	39.91	35.06	−1.88	0.06
	Part-time	11.75	12.80		
	Hourly	2.51	3.35		
	Volunteer^b^	0.78	0.92		
	Unemployed	5.60	8.54		
	Retired	29.25	20.73		
	Household^c^	3.51	7.93		
	Apprenticeship	1.18	3.05		
	Student	5.10	7.32		
Household Income^d^	< 750 €	3.98	3.76	−0.21	0.82
	750 to < 1250 €	15.21	12.23		
	1250 to < 2000 €	26.82	32.29		
	≥ 2000 €	54.01	51.72		
Religion^e^	Protestant	38.77	17.99	−2.50	0.01**
	Catholic	31.25	35.06		
	Muslim	0.18	17.68		
	Other	0.46	9.76		
	No religion	29.09	18.77		

Note ^a^ separated but still legally married; ^b^ Volunteer includes those on parental leave; ^c^ Household refers to those not working but not unemployed; ^d^ Household income per month; ^e^ 12 participants did not respond, i.e. 9 German natives and 3 migrants

### Measures

#### Migration

In line with Beutel et al. [10] a variable distinguishing German natives from migrants was generated in accordance with the German micro census definition. The variable combines information on participants’ citizenship and birthplace of both mother and father. Therefore, in this study, the term ‘migrant’ is used to identify first- and second-generation migrants. First generation migrants include non-German citizens who migrated to the Federal Republic of Germany after 1949, while second-generation migrants are all non-German citizens born in Germany and all citizens born in Germany with at least one migrated parent.

#### Perceived stress scale (PSS-10)

The German version of the PSS-10 was translated and standardized by Klein et al. [23]. Respondents report the degree to which situations in one’s life have been unpredictable, uncontrollable and overloaded in the past month on a 5-point Likert scale (0 = never, 1 = almost never, 2 = sometimes, 3 = fairly often, 4 = very often). Scores for the four positively stated items (Items 4, 5, 7, 8) are reversed.

### Statistical procedure

Analyses were computed using R v3.5.0 [26] and associated packages of *Lavaan* [27]*, psych* [28]*, GPArotation* [29] and *MBESS* [30]. Descriptive statistics were tabulated by migrant status (German native and migrant) to provide a breakdown of the nationally representative sample. T-tests assuming equal variance on key sociodemographic variables were calculated to determine the comparability of the two samples.

There were few missing data on the PSS, four persons did not answer any item and were dropped completely. In the remaining group, two persons left three items unanswered, three persons two items, and 55 persons one item. Also, across the sub-samples there were few missing item values, Item 7 had the highest with 12 missing values in the native sample (0.05%), all together the missing PSS item values do not exceed 2.4% in the native sample and 4.2% in the migrant sample. Hardt et al. [31] suggests as a rule of thumb with less than approximately 5% missing data, no measures such as multiple imputation should be performed. In response to missing data and non-normality, maximum likelihood (ML) estimation with robust standard errors and Satorra-Bentler scaled test-scores was deemed the most appropriate method [32, 33]. Cronbach’s alpha was calculated on pair wise covariances, for correlations and EFA no adjustments were made.

A single EFA using maximum likelihood estimation with oblimin rotation on the migrant sample was completed to determine the appropriate PSS-10 factor structure [34]. Separate CFAs were calculated on the native and migrant samples to test the established two-factor structure [18, 23], followed by a multigroup confirmatory factor analysis (MGCFA). The negatively worded items form factor one, while positively worded items form factor two. Goodness of fit was evaluated based on Satorra-Bentler [32] adjusted chi-square (χ^2^), standardized root mean square residual (SRMR), comparative fit-index (CFI), Tucker-Lewis Index (TLI), as well as the root mean square error of approximation (RMSEA) and its 90% confidence interval (90% CI). Higher values of CFI and TLI (> 0.95) indicate a better model fit, while SRMR and RMSEA values below 0.08 are recommended [35].

In line with Milfont and Fischer [36], measurement invariance of the PSS-10 between the native and migrant samples was tested using four hierarchical models: (1) configural, (2) metric, (3) scalar, and (4) strict. Model (1) tests that the PSS-10 two-factor structure is invariant in both groups. Model (2) holds factor loadings equal across groups, followed by model (3) that additionally constrains item-intercepts. Lastly, model (4) constrains factor loadings, intercepts and error variances between native and migrant samples. Measurement invariance was evaluated by changes (Δ) in goodness of fit indices including: Δχ^2^, ΔCFI, ΔTLI, ΔSRMR and ΔRSMEA. When sample sizes are unequal, as is the case in this study, Chen [37] recommends the following cutoff criteria for testing levels of invariance: ΔCFI ≥ − .005, ΔRSMEA ≤ .010 or a ΔSRMR ≤ .025 (≤ .005 for intercept and residual invariance) indicate invariance.

Reliability of the German version PSS-10 was evaluated via Cronbach’s alpha (α), omega (ω), and associated 95% confidence intervals. Alpha and omega were calculated using a 1000-repetition bootstrap method to obtain percentile confidence intervals. Higher values of omega and alpha (.70 and greater) reflect stronger internal consistency [38–40]. Means of individual items were examined between German natives and migrants via t-tests and Cohen’s d effect size. Cohen’s d effect size tests the standardized mean difference for each item between German natives and migrants. Cohen’s d values are evaluated as irrelevant (d < .20), small (.20 ≤ d < .50), medium (.50 ≤ d < .80), and large (d ≥ .80) [41]. Lastly, PHS and PSES scores were compared using t-tests and associated Cohen’s d effect size between migrants and natives as well as between first- and second-generation migrants.

## Results

### Sample descriptive statistics

The nationally representative sample included *N* = 2523 participants, of which *n* = 328 participants had a migration background and *n* = 2195 were German natives. Migrants were significantly younger than German natives by approximately three years (*t*_2525_ = 3.23, *p* < .001). The two sub-samples did not statistically differ based on sex (χ_1_^2^ = .52, *p* = .46) or household income (χ _3_^2^ = 4.96, *p* = .17). First-generation migrants account for *n* = 76 participants, while *n* = 252 are considered second-generation migrants. The two generational groups did not statistically differ based on sex, age, or household income.

### Descriptive item analysis

Table 2 displays the mean (M), standard deviation (SD), and the corrected item-rest correlation (r_it_) for each PSS item separated by sub-samples. T-tests for each item compared the means of German natives to migrants showing that six out of the ten items were significantly different with a *p*-value of .05 or lower. However, Cohen’s d effect size reports that only item 3 has a small effect size where migrants differ from natives by more than .20 standard deviations, while all other items have an irrelevant effect size.

**Table 2:** PSS item descriptives

Item		Natives	Migrants	Cohen’s d	t-test	*p*-value
Factor 1 PHS						
1	M	1.19	1.23	−0.051	−0.87	0.381
	SD	0.92	0.98			
	r_IT_	0.55	0.59			
2	M	0.88	1.01	−0.141	−2.38	0.017**
	SD	0.94	0.98			
	r_IT_	0.69	0.65			
3	M	1.38	1.65	−0.266	−4.50	< 0.001**
	SD	0.98	1.07			
	r_IT_	0.59	0.52			
6	M	1.00	1.10	−0.112	−1.89	0.057
	SD	0.93	0.94			
	r_IT_	0.67	0.69			
9	M	1.59	1.71	−0.122	−2.06	0.039*
	SD	1.02	0.98			
	r_IT_	0.54	0.46			
10	M	0.99	1.10	−0.105	− 1.77	0.076
	SD	0.98	1.05			
	r_IT_	0.73	0.70			
Factor 2 PSES						
4	M	1.37	1.52	−0.143	−2.42	0.015**
	SD	1.10	1.10			
	r_IT_	0.69	0.62			
5	M	1.51	1.63	−0.119	−2.01	0.044*
	SD	1.02	1.00			
	r_IT_	0.69	0.65			
7	M	1.40	1.52	−0.110	−1.85	0.063
	SD	1.07	1.05			
	r_IT_	0.67	0.71			
8	M	1.14	1.28	−0.144	−2.43	0.015**
	SD	0.96	0.97			
	r_IT_	0.72	0.65			
PHS	M	7.01	7.76	−0.172	−2.89	0.003***
	SD	4.34	4.42			
PSES	M	5.41	5.95	−0.157	−2.62	0.008***
	SD	3.46	3.36			

Note: Sample Size (*N*), mean (*M*), standard deviation (*SD*), item-rest correlation (*r*_*IT*_). Sample size for items range between 2183 and 2195 for natives and 324–328 for migrants. Sig. difference shows the p-value of a two-tailed t-test and significance value * *p* < 0.1, **p < 0.05, *** *p* < 0.01. Effect size reports the Cohen’s d. Degrees of freedom for the t-tests varied between 2506 and 2518 due to missing data

### Factor analyses

An EFA tested the German version PSS-10 factor solution with the migrant sample. Results showed a two-factor solution where 56% of the variance was explained by Factor 1 (PHS) with loadings from .55–.78; while Factor 2 (PSES) explained 44% of the variance with loadings ranging between .68–.83.

Model fit indices of the individual CFAs for German natives and migrants are reported in Table 3. The Sattora-Bentler scaled χ^2^ test is significant at the 1% level for both natives and migrants, indicating poor model fit. As χ^2^ is highly sensitive to sample size and non-normality of data, additional scaled indicators were evaluated for model fit. In both samples, SRMR and RMSEA < .08 and CFL > .95 all indicate good model fit and while TLI is not greater than .95, it is close with .93. Overall, the two-factor PSS fits the data in both native and migrant samples.

**Table 3:** Individual & Multigroup CFA model fit

	*N*	χ^2^ (df)	CFI	TLI	SRMR	RMSEA	90% CI
Native	2146	446.21(34)***	0.947	0.929	0.053	0.082	0.076–0.089
Migrant	317	81.44(34)***	0.945	0.928	0.060	0.077	0.056–0.099
Configural		505.17(68)***	0.947	0.929	0.049	0.082	0.075–0.088
Metric		521.65(76)***	0.946	0.937	0.051	0.077	0.071–0.084
Scalar		547.53(84)***	0.946	0.942	0.051	0.074	0.068–0.080
Strict		490.40(94)***	0.945	0.948	0.052	0.070	0.064–0.077

Note: robust estimates, ***sig. *p* < 0.001

The hierarchical measurement invariance models were computed with robust maximum likelihood method. Measurement invariance was evaluated by the change (Δ) in goodness of fit indices. Table 4 shows the changes in robust model fit between the invariance models. Although χ^2^ values violate invariance assumptions, in testing the loading, intercept and residual invariance, ΔCFI exceeds the −.005 threshold, ΔSRMR and ΔRSMEA are lower than the .025 and .010 cutoff, indicating invariance. Thus, the two-factor PSS-10 is a strict invariant measure between German natives and migrants.

**Table 4:** Change in goodness of fit MGCFA

	Δχ^2^ (df)	ΔCFI	ΔTLI	ΔSRMR	ΔRMSEA
Metric	8.8(8)	0	0.007	.001	−0.004
Scalar	16.7(8)	−0.001	0.005	.001	−0.003
Strict	11.5(10)	0	0.006	0	−0.004

Note: showing the difference of the model from the model before (metric = metric-config)

### Reliability

As both the EFA and CFA results show and confirm a two-factor structure, Cronbach’s alpha and omega for each factor was calculated, higher values of omega and alpha indicate good internal consistency. Perceived helplessness (PHS-factor 1) showed a good internal consistency in both the native sample (α = .85 SD = .25 95% CI = .83–.85; ω = .85 SD = .01 95% CI = .84–.86) and in the migrant sample (α = .83 SD = .32 95% CI = .80–.86; ω = .83 SD = .02 95% CI = .80–.86). Similarly, PHS Perceived self-efficacy (PSES-factor 2) showed good internal consistency for the native (α = .85 SD = .34 95% CI = .84–.87; ω = .85 SD = .01 95% CI = .84–.87) and migrant (α = .83 SD = .37 95% CI = .79–.86; ω = .83 SD = .02 95% CI = .79–.87) samples.

### Perceived stress of migrants

In testing the PSS sub-scale scores between natives and migrants, natives scored significantly lower on both the PHS (t_2494_ = − 2.89, *p* < .01) and the PSES (t_2485_ = − 2.63, p < .01). Cohen’s d effect size for PHS (d = −.17) and PSES (d = −.16) show irrelevant effects although the t-tests are significantly different. In comparing first- and second-generation migrants, first-generation migrants score significantly lower on the PHS scale than second-generation migrants (t_320_ = 1.67, *p* < .05), albeit a Cohen’s d of only .22 reveals a small effect size. No significant difference of the PSES was found.

## Discussion

Lee [15] called for validating the PSS in representative populations and Klein et al. [23] responded by testing the German version PSS-10 in a representative German population. The current study took the analysis in the same sample one step further by testing whether the German version PSS-10 is also a reliable and valid measure of perceived stress in a migrant sample.

Overall the PSS-10 maintains a two-factor structure that is strictly invariant between German natives and migrants, it additionally shows good internal consistency as found in other diverse populations, see review [15]. Omega and Cronbach’s alpha for the PSS-10 sub-scales ranged from .83 to .85 thus supporting the reliability of the German version PSS-10. Migrants reported higher scores on both PSS sub-scales, indicating higher levels of perceived helplessness as well as lower levels of perceived self-efficacy since the latter scale is reversed. However, by taking into account Cohen’s d effect size, there is no relevant effect between the two samples on PHS or PSES.

When comparing first- and second-generation migrants, perceived self-efficacy showed no significant difference, however, first-generation migrants report significantly lower perceived helplessness scores with a small effect size. Although this finding is modest, it is at odds with previous literature, which generally show that first-generation migrants report higher rates of mental distress compared to natives and second-generation migrants [10, 11]. Factors of language skills, unemployment, perceived discrimination, integration or acculturation, and cultural barriers often reveal differences between first- and second- generation migrants [10, 11, 42]. It is important to note the sample size of the current study, as first-generation migrants make up a very small proportion of total migrant sample (*n* = 76). While the second-generation sample may still emulate representative characteristics (*n* = 252), it is unlikely that 76 migrants represent the linguistic, religious, and cultural diversity of the first-generation migrants residing in Germany. For many first- and second- generation migrants, the cultural background plays an important role. For instance, migrants of Turkish origin report higher rates of depression, anxiety, and suicidal ideation compared to Polish origin migrants and German natives [10]. Similarly among first-generation migrants, Tibubos et al. [11] illustrate that those with a Turkish origin report higher rates of mental distress symptoms compared to other geographical origins. Self- and group- attribution as migrants are additional factors that may influence not only differences between migrants and native Germans, but also differences between first- and second-generation migrants. Nesterko et al. [43] explored attribution as a migrant as a predicting factor of PTSD, depression and anxiety, finding that many second-generation migrants do not self-attribute as a migrant. These types of factors are likely to influence perceptions of discrimination and ultimately levels of perceived stress.

With respect to the PSS-10 factor structure, the ongoing one- or two-factor debate led this study to test the scale factor structure using an EFA specifically in the migrant sample. Results showed a two-factor solution with good factor loadings, thus reflecting the established two-factor structure presented by Hewitt et al. [16] and Roberti et al. [17], where negatively worded items represent factor 1 (*perceived helplessness*) and positively worded items make up factor 2 (*perceived self-efficacy*). Item-rest correlation values are generally consistent across the native and migrant samples, emphasizing that items fit to the total scale similarly across the samples. Separate group-specific CFA and combined MGCFA model fit indices were in line with the recommended values of Schermelleh-Engel, Moosbrugger, & Müller [35]. Therefore, the current two-factor structure shows adequate model fit in the native and migrant samples.

As for measurement invariance, the two-factor model is strictly invariant between German natives and migrants. Given that χ^2^ is sensitive to large sample sizes and violations of normality [33, 36, 37], with a total sample of *N* = 2523, it was expected to be significant showing poor model fit. Chen [37] discusses similar biases caused by large or unequal sample sizes in evaluating measurement fit indices; as with increasing sample size, the standard deviations of fit indices will decrease leading to higher chances of rejecting the model. Additionally, unequal samples may conceal non-invariance as the larger group will dominate calculations of many fit indices, leading to small changes between invariance models [37]. This is likely the case with the current samples, ultimately showing very low changes in all fit indices across models. Even so, considering that the data violates normality and has a large, particularly unequal, sample size, ΔCFI, ΔSRMR and ΔRSMEA still meet strict invariance.

Despite this study’s support of the two-factor structure and results of others showing independent explanatory power of the individual factors [24], the general PSS total scale also consistently demonstrated good Cronbach alpha values offering a simplified interpretation of perceived stress [15]. Klein et al. [23] report good internal consistency for the German version PSS-10 total scale (Cronbach alpha = .84). Cohen and Williamson [14] challenge the content relevance of the second factor and Reis et al. [24] rather argue for a bifactor solution where each factor is content relevant. Given these contradicting results it is difficult to recommend using one single solution for all analyses – rather, while many studies find two factors, the total PSS score may still be appropriate. Therefore, as Reis et al. [24] elaborate, the decision of implementing the total score or sub-scales depends highly on the research focus, furthermore one must take caution when interpreting means of the total score as well as covariances or correlations with other concepts.

Although the PSS-10 was not intended to operate as a diagnostic measure [44], the scale may signal early signs of mental distress for a broad range of stressful living conditions. Perceptions of helplessness and self-efficacy are key factors in stress processes and stress regulation [25]. In clinical settings, it could be used to adjust stress management strategies, understand worsening symptoms due to stress, and facilitate the development of coping skills [13, 17, 18]. Schneider et al. [25] support the use of the German PSS-10 in clinical populations as a first-screening instrument. Potential clinical applications of the German PSS-10 are relevant for residents with a migration background as it may identify individuals with heightened levels of stress, indicating potential risk factors for other mental health concerns.

Overall, the German version PSS-10 is a valid, strictly invariant and reliable measure of perceived stress among German natives and residents with a migrant background. These results are valuable as implications of the PSS-10 can aid German health officials as well as stress researchers to understand stress in migrant populations in Germany.

### Strengths & Limitations

To the authors’ knowledge this is the first study to evaluate and compare the psychometric properties of the German version PSS-10 between native Germans and a migrant sample. One major strength of this study stems from the total sample size and quality of the nationally representative data. Of the many studies that translated and standardized the PSS-10, most studies used university student samples [15] or very specific samples [20–22].

Although results of the MGCFA show acceptable fit and strict measurement invariance, the stark difference in migrant (*n* = 328) and native (*n* = 2195) sample size should raise caution. As mentioned above, there are associated biases that come with large and unequal samples [37]. In ideal circumstances, factor analyses should further separate first- and second-generation migrants in comparison to German natives. Previous studies have presented stark differences between first- and second-generation migrants [10, 11] where second-generation migrants are more comparable to German natives and seen as “adjusted” rather than first-generation migrants [10]. Therefore, by merging both groups together there is a potential weakened effect of migration status in comparison to German natives. However, due to small sample size, factor analysis on migrant groups was deemed inappropriate [33, 34]. This small sample size, specifically of first-generation migrants, is unlikely to represent the diversity of first-generation migrants in Germany.

In a similar vein, language skills required to complete the interview process may have led to a sampling bias towards first-generation migrants with adequate German language. Additional cultural aspects need to be considered such as religious backgrounds or country of origin, as they are expected to have an influence on not only the outcome of scales but also the understanding of scales [8, 9, 11]. Lastly, the migrant sample was significantly younger (by 3 years) compared to the German natives, which may also impact the validity of the scale, as age is continuously shown as an influencing factor of perceived stress [19, 21, 23].

### Future research

Future research must critically evaluate the PSS-10 two-factor structure. With continued support for two-factors, the research community must then adapt to reporting the two factors separately rather than the total score value. Reis et al. [24] recommends that researchers understand their use of the scale in determining whether to use it with a one- or two-factor structure.

In efforts to advance stress research in migrant populations, the PSS-10 should be further tested for its psychometric properties among first- and second-generation migrant samples, as the two migrant groups may experience stress differently. Additionally, taking into account one’s migration status (e.g. asylum seeker, refugee, economic migrant, international student etc.) would expand the current understanding of how various migrant classifications influence not just one’s perceived stress but also the validation of the PSS-10. Representative studies would also benefit from more detailed accounts of migrant group representation and should therefore include all migrant groups within a country in data collection strategies. Lastly, future research needs to not only focus on acute perceived stress but also the more dangerous chronic forms of stress. Thus, testing the psychometric properties of chronic stress scales such as the Trier Inventory of Chronic Stress (TICS) in refugee and migrant populations would further advance stress research.

## Conclusion

This study tested the validity and reliability of the two-factor structure of the German version PSS-10 and measurement invariance in native and migrant subsamples. The key conclusions are: (1) the German version PSS-10 is suitable for comparing German residents with and without a migration background. (2) Despite good internal consistency for the total scale, the PSS-10 measures two aspects: (a) perceived helplessness and (b) perceived self-efficacy. Future research would profit from analyzing the two subscales separately, not only using the total score.

## Data Availability

The data used in this manuscript is not publicly available but may be available from Prof. Brähler upon reasonable request.

## Abbreviations

PSS: Perceived Stress Scale
PSES: Perceived Self-Efficacy
PHS: Perceived Helplessness
EFA: Exploratory Factor Analysis
MGCFA: Multi-Group Confirmatory Factor Analysis
Baua: Bundesanstalt für Arbeitsschutz und Arbeitsmedizin [Federal Institute for Occupational Safety and Health]
WHO: World Health Organization
MI: Measurement Invariance
ML: Maximum likelihood
CFA: Confirmatory Factor Analysis
Χ^2^: Chi-square
SRMR: Standardized root mean square residual
RMSEA: Root mean square error of approximation
CFI: Comparative Fit Index
TLI: Tucker-Lewis Index
